# Transfer of an interprofessional emergency caesarean section training program: using questionnaire combined with outcome data of newborn

**DOI:** 10.1007/s00404-020-05617-z

**Published:** 2020-07-13

**Authors:** Markus Flentje, Hendrik Eismann, Maike Höltje, Vera Hagemann, Lars Brodowski, Constantin von Kaisenberg

**Affiliations:** 1 Department of Anaesthesiology and Intensive Care Medicine, Hannover Medical School, Carl-Neuberg-Str. 1, 30625 Hannover, Germany; 2 Department of Anaesthesiology and Intensive Care Medicine, Winsen Hospital, Friedrich-Lichtenauer-Allee 1, 21423 Winsen, Germany; 3 Faculty of Business Studies and Economics, University of Bremen, Enrique-Schmidt-Strasse 1, 28359 Bremen, Germany; 4 Department of Obstetrics and Gynecology, Hannover Medical School, Hannover Medical School, Carl-Neuberg-Str. 1Carl-Neuberg-Str. 1, 30625 Hannover, Germany

**Keywords:** Emergency caesarean section, Training, Interdisciplinary collaboration, Simulation

## Abstract

**Puprose:**

An emergency caesarean section is a potentially life-threatening situation both for the mother and the newborn. Non-technical skills can be improved by simulation training and are necessary to manage this urgent situation successfully. The objective of this study was to investigate, if training of emergency caesarean section can be transferred into daily work to improve the outcome parameters pH an APGAR of the newborn.

**Methods:**

In this pre-post study, 141 professionals took part in a training for emergency caesarean section. Participants received a questionnaire, based on the tools “Training Evaluation Inventory” and “Transfer Climate Questionnaire” 1 year after training. Outcome data of the newborn were collected from the hospitals information system.

**Results:**

Except the scale “extinction”, Cronbach’s alpha was higher than 0.62. All scales were rated lower than 2.02 on a 5-point Likert Scale (1 = fullest approval; 5 = complete rejection). “Negative reinforcement” was rated with 2.87 (SD 0.73). There were no significant differences in outcome data prior. The questionnaire fulfils criteria for application except the scale “extinction”.

**Conclusion:**

The presented training course was perceived as useful by the professionals and attitudes toward training were positive; the content was positively reinforced in practice 1 year after training. Parameters of the newborn did not change. It is conceivable that other outcome parameters (e.g. posttraumatic stress disorder) are addressed by the training. The development of relevant outcome parameters for the quality of emergency sections needs further investigation.

## Introduction

The emergency caesarean section represents the escalation of an obstetric emergency. Because a vital risk to the mother and/or the child is suspected, the team must carry out the birth of the child under extensive pressure of action. All members of the multidisciplinary team of obstetricians, midwives, anaesthesiologists and nurses will need to complete their job-related work very quickly and in strict chronologic arrangement. As a temporal quality criterion for the assessment of the emergency caesarean section, the decision-to-development interval (DDI) is taken. The recommendation to the time frame conditions in Europe is different. For examples, the NHS (National Health Service) in the UK recommends a DDI of 30 min, in case of an immediate threat to the life of the mother or the foetus. Otherwise a DDI of 75 min should be sufficient. In Germany, the guidelines are specified by the “Deutsche Gesellschaft für Gynäkologie”. These stipulate that all structural and procedural requirements must be in place that a DDI of 20 min can be kept. It is mentioned that this time still seems too long in individual cases, so all measures should be checked to shorten this time furthermore [1]. Heller et al. were able to show retrospectively with almost 40,000 cases that there are better APGAR scores in newborn delivered by emergency caesarean section at shorter DDI [2]. The continuing value of the APGAR score is used for the assessment of newborn for a long time [3]. A second criterion for the outcome of the newborn is the umbilical pH. Low arterial cord pH is associated with increased neonatal mortality and hypoxic ischaemic encephalopathy [4].

From an industrial psychology point of view, the professionals of the delivery room, in particular, the situation of an emergency caesarean section, fulfils the general conditions of a high responsibility team (HRT). Death as a result of decisions made, the irreversibility of many therapeutic actions and time pressure are some characteristics of this work environment [5]. An analysis of Germany’s critical incident reporting system (CIRS) showed frequent potential adverse events in emergency caesarean sections [6].

In the UK with its long tradition of systematic evaluation of obstetrics, the reports call for communication and crisis resource management courses [7]. To be successful in this work environment, other HRT such as aviation has shown that a training of non-technical skills can reduce errors and adverse events [8]. Task management, teamwork, situational awareness and decision-making are categories of non-technical skills [9]. Improvements in anaesthesiologists’ non-technical competencies by simulator based training have previously been shown [10].

In Germany, there is currently no obligation for practical training in multiprofessional delivery room teams. Nevertheless, there are various local projects, that differ in training group, duration and training objectives [11–13]. Our working group developed a team training to the subject “emergency caesarean section” which is also realistically applicable in the German health care system, by 4 h’ duration [14] and is evaluated for subjective competence gain [15].

Training for medical professionals should not be an end in itself. The use of high training costs through planning and staffing has to be evaluated. Also, the question of whether there is harmful training with negative patient outcome must be considered. Kirkpatrick suggested the best known and most rigorous form of evaluation at four outcome levels [16]. After reactions, learning and behavioral change of participants, outcome results are the highest level.

The aim of this study was to evaluate the multiprofessional training program for emergency caesarean section regarding to Kirkpatrick’s level “reactions”, “behavioural change” and “outcome” by questionnaire and outcome data. The hypothesises were (1) the questionnaire meets the quality criteria, (2) the results show positive reaction and transfer performance of the participants depending on the trained professional group and (3) outcome parameters of newborn have changed by the intervention of training.

## Methods

### Study design

This study was pre-post study with participants of the HAINS (Hannoeverscher Anaesthesie- Intensiv- und Notfall- Simulator) Safety® education programme at Hannover Medical School. The program focuses on multiprofessional training of in-hospital emergency situation. The study was approved by the ethics committee of the Hannover Medical School (no. 7511).

### Setting and population

The participants were employees of a university hospital. The departments Obstetrics and Genecology (tertiary referral centre, dates of birth Table 1) and the department Anaesthesiologie participated in the training. The participants were defined by the respective department heads in a duty roster. In total, 15 theatre nurses, 46 anaesthetic nurses, 23 obstetricians, 31 midwifes and 26 anaesthesiologists (in total 141) were trained in 16 training courses. The participation was planned by the supervisors. At least, 80% of the delivery room staff of each department was trained. Participation in the questionnaire was voluntary, anonymous and participation could be withdrawn at any time without giving reasons.

**Table 1:** Structural data of the clinic 1 year prior and 1 year after training

	Prior training (T0)	After training (T1)
Number of emergency sections	54	37
Number of caesarean sections	919	809
Number of births over all	2982	3096
Decision-to-development interval (DDI)		
< 3 min	1	0
3–10 min	48	35
11–20 min	5	2

The training period of 3 month was excluded

### Intervention

The course “HAINS Sectio” was designed for multiprofessional teams of a delivery room. The training has two goals: first, the standard operation procedure (SOP) of an emergency caesarean section from indication to skin incision should be practiced, second, in the sense of crisis resource management training, the communication of relevant information, task management and decision-making should be improved. The course started with a lecture on non-technical skills, indications of an emergency caesarean, anaesthetic specifics and the local SOP. After a familiarisation with the simulation environment, each participant participated in two scenarios with all involved occupational groups. Each scenario started in a delivery room with a midwife and a patient simulator (SimMom, Laerdal Medical, Norway). After recognition of a life-threatening situation, the decision to perform an emergency caesarean was made. The other professional groups had to be summoned, the simulator was transferred to the simulated operation theatre and the scenario continued running until the skin incision. After each scenario, a video debriefing according to specifications of the TeamGAINS [17] structure with focus on non-technical skills was performed. The 4 h training course ended after a feedback round.

### Questionnaires

Selected scales of two valid questionnaires were applied. We focused only on the most relevant scales for analyzing the hypotheses to keep the questionnaire short and to obtain a realistic number of answerable questions during the routine work. The focus of the study was on the usefulness and transfer of knowledge.

The questionnaires registered professional group and gender of the participants. From the Training Evaluation Inventory (TEI), two scales (5-point Likert Scale from 1 to 5; 1 = fullest approval; 5 = complete rejection) were taken. This instrument was chosen as it was validated in a German language and German culture sphere [18]. The two scales of interest were “perceived usefulness” and “attitude towards training” and covered training outcomes on the first level of Kirkpatrick’s four levels of training evaluation [16]. Internal consistency of the scales is perceived usefulness (*α* = 0.89) and attitude towards training (*α*  = 0.81) [18].

From the Transfer Climate Questionnaire, already used in other studies evaluating crisis resource management interventions in high responsibility teams (HRT) [19], five scales (5-point Likert Scale from 1 to 5; 1 = fullest approval; 5 = complete rejection) were taken. The scales of interest covered “cues”, “reinforcements” and “extinction possibilities” and represented Kirkpatrick’s level three of evaluation (behavior). Internal consistency of the subscales inherited from the literature were social cues (*α*  = 0.91; 2 items), task cues (*α*  = 0.26; 2 items), positive reinforcement (*α*  = 0.81; 3 items), negative reinforcement (*α*  = 0.59; 3 items) and extinction (*α*  = 0.78; 2 items). All 19 items are completely shown in “Appendix 1”.

One year after each course, the participants got a link to the online questionnaire by email. Each questionnaire was conducted using the online survey platform SurveyMonkey (SurveyMonkey, San Mateo, USA). After 7 days, the participants received a reminder to complete the survey by email.

### Outcome data

The analyzed cases were exported from medical information system using the keyword “emergency caesarean” and by selection of the desired time period. The parameters APGAR, pH and base excess of the newborn were gathered by the hospitals documentation system. These data were routinely collected as part of a regional quality assurance program. The numbers of births, caesarean sections, emergency caesarean sections, as well as DDI were also gathered by the hospital information system. The observation period was 1 year before and 1 year after training courses. The time period of the courses (3 month) was ignored—data were not gathered.

### Statistical analysis

Demographic and survey data were analyzed in a descriptive manner. The reliabilities of the scales were determined by Cronbach’s alpha. The normal distribution of the values was reviewed using the Kolmogorov–Smirnov test.

To test differences in training evaluation, we conducted a one-way ANOVA.

To test hypothesis (c), a *t* test for independent samples was conducted. We assumed *p* < 0.05 as being statistically significant. Cohen’s d was calculated as effect size. All calculations were made using SPSS Statistics 24 (IBM Corporation, USA).

## Results

Overall, the survey was completed by 83 participants. Due to incomplete data, 74 questionnaires were included into the analysis (overall response rate 60%). Forty-six (55.4%) participants were female, 28 (33.7%) male. Represented by professional group, 10 theatre nurses, 24 anesthetic nurses, 11 obstetricians, 16 midwifes and 13 anesthetists completed the questionnaire.

### Reliability of the scales

To test hypothesis, (a) Cronbach’s alpha was analyzed (Table 2). The scales “perceived usefulness”, “attitude towards training”, and “task cues” showed good reliability. The subscales “social cues” (0.62) and “positive reinforcement” (0.64) showed a satisfactory reliability. By removing the item “colleagues ridicule the use of methods in training”, Cronbach’s alpha of the scale “negative enforcement and punishment” could be increased from 0.48 to 0.71. The scale “extinction” shows a very low reliability for further interpretation (0.31).

**Table 2:** Reliability of the scales with Cronbach’s alpha

Item	Cronbach’s alpha	ANOVA
Perceived usefulness (4 items)	0.86	*F*(4,69) = 0.728, *p* = 0.576
Attitude towards training (3 items)	0.84	*F*(4,69) = 0.525, *p* = 0.718
Social cues (2 items)	0.62	*F*(4,69) = 1.544, *p* = 0.199
Task cues (2 items)	0.75	*F*(4,60) = 0.253, *p* = 0.907
Positive reinforcement (3 items)	0.64	*F*(4,69) = 1.180, *p* = 0.327
Negative reinforcement and punishment (3 items) Removed item: “Colleagues ridicule the use of methods in training”	(0.48) 0.71	*F*(4,69) = 1.335, *p* = 0.266
Extinction (2 items)	0.31	*F*(4,60) = 0.770, *p* = 0.548

By removing an item the scale “negative enforcement and punishment” could be increased. The scale “extinction” shows a very low reliability for further interpretation

### Evaluation by professional groups

**Fig. 1:**
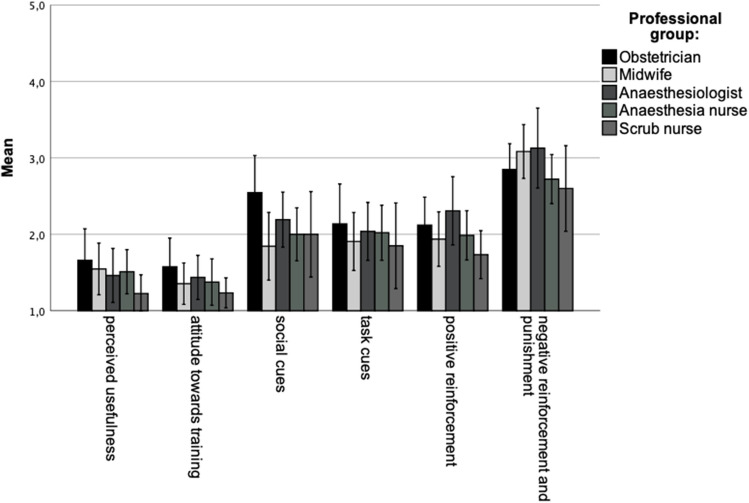
Average and standard derivation of training evaluation. There was no statistically significant difference within the professional groups

To evaluate the attitude and reaction towards the training as well as the transfer of the training in the working field, the arithmetic mean was calculated within each scale. The results were: perceived usefulness 1.49 ± 0.61, attitude towards training 1.39 ± 0.56, social cues 2.08 ± 0.78, task cues 1.99 ± 0.75, positive reinforcement 2.02 ± 0.67 and negative reinforcement and punishment 2.87 ± 0.73. Values of the professional groups are shown in Fig. 1. There were no significant differences in the evaluation of the training course by the individual occupational groups.

### Outcome of the newborn

To test hypothesis (c), the change in APGAR, pH and base excess in newborn after emergency caesarean, scores from the newborn 1 year before (T0) and 1 year after the training (T2) were collected. One record in group T1 was incomplete and was excluded for further analysis. Table 1 shows count of emergency sections and DDI in the investigated time periods.

The data of APGAR, pH and base excess are shown in Table 3. All data were normally distributed, as assessed by the Kolmogorov–Smirnov test.

**Table 3:** Outcome date of the newborn

	Prior training (T0)	After training (T1)	*p*	Cohen’s *d*
	*n* = 54	*n* = 36		
APGAR 1 min	6.83 ± 2.15	5.97 ± 2.57	0.89	0.36
APGAR 5 min	8.39 ± 1.71	7.75 ± 2.21	0.13	0.32
APGAR 10 min	9.2 ± 1.47	8.78 ± 1,97	0.24	0.24
pH	7.16 ± 0.11	7.15 ± 0.15	0.72	0.08
BE	− 8.49 ± 4.55	− 8.22 ± 6.82	0.82	0.05

With a small number of cases, there is no significant difference within the groups. The effect size is very small

## Discussion

We developed a team training to the subject of “emergency caesarean” that is realistically applicable in the framework of the German health care system [14]. The aim of the study was to evaluate the training in regard to the levels of Kirkpatrick [16].

To test hypothesis (a), the scales require qualitative consideration. Almost all scales showed acceptable to good values and can be applied to evaluate our developed training. It is noticeable that all scales describing people’s collaboration have slightly lower values of Cronbach’s alpha. The scales can be used excluding the scale “extinction”, which does not fulfil the criterion of further use. In our interpretation, this is a result of the complex situation of a multiprofessional healthcare. The terms “colleague” and “supervisor” can be interpreted differently in the questionnaire (question: supervisors do not care if I use the skills learned in training, as long as I get the job done). The regular hierarchy in this teams is not comparable to other organization, as each individual department has full responsibility for its actions. As example, a conflict of interest occurs in the situation of an emergency caesarean, if the anaesthesiologist needs to preoxygenate the patient prior induction and the obstetrician wants an instant beginning of anaesthesia. The manner of leadership in obstetric emergencies is a widely discussed topic [20].

The second question of scale extinction deals with the question of the applicability of the training content (question: have so little chance to use some of the skills learned since training, that I probably could not perform them now). The method of “Climate for Transfer” Model was primarily developed in the context of a training for flight engineer [21]. In this working environment, professionals are regularly confronted with recurring situations. The character of the emergency event is accompanied by the fact that the confrontation of the participant with the situation varies. So, the question is not suitable for the entire group of participants. Therefore, we recommend checking the necessity of the scale extinction and possibly developing new items.

To test hypothesis (b) (positive reaction and transfer to clinical practice), we applied the valid questionnaires. The rating on all individual scales is good to very good. If the employees are recognized as experts in the situation, the course is rated excellent in regard of usefulness and transfer (Kirkpatrick level 3).

In Sweden, it could be shown that after simulation-based team trainings, the number of nurses quitting their jobs and nurse assistants’ time sick leave was reduced [22]. In the same study, the safety attitudes questionnaire (SAQ) Index [23], one of the most validated “safety climate” measuring tools, increased. With current staff shortage in German hospitals [24], a simulation course offered to the employees, could be an important argument for staff retention. After our work confirmed the transfer to the clinic, the increase in SAQ and effects on the availability of professionals could be a parameter for further studies.

There are no differences in the assessment of the training between the professional groups. This result justifies the situation emergency cesarean to be equally relevant for the entire team.

The lowest rating for the transfer, indicates the value of “negative reinforcement”. Introduction of SOP requires continuous efforts and the value should be better after continuous training over time. Implementation and compliance with SOP are challenging [25], as a procedure can be successful in a variety of ways. In the context of other critical situations, it is known that SOP improves patients care [26].

In our study, we were unable to show any impact of training on outcome parameters of the newborn (hypothesis c). The effect sizes were very low. Significant results could only be expected with a higher number of cases. This observation is in-line with the study by Heller et al., which has integrated 40,000 cases into account [2]. Our number of cases was limited, as we observed existing emergency caesarean. The observation period should intentionally not be extended beyond 1 year, as we assumed an eased training effect as shown in with other training contents [27]. Heller reported an effect at the 10- and 20-min mark, which we could not show in our setting. As a university hospital with about 3000 births a year, we always have an anesthesia team available. So, the DDI time was almost always shorter than 10 min (Table 1) before training and the mean child outcome data in our study can be classified on the best cohort of Heller’s scale. We interpret the structure of the authors own clinic regarding newborn’s outcome as being good prior training. This study underlines that a training effect with the known parameters can only achieved through large numbers of cases in different level of care hospitals.

Studies show that a considerable financial outlay is made for the posttraumatic care of woman and that negative experiences of a delivery have a negative effect on mother–child relationships. In our study group, we evaluated a subjective quality catalog for women which needed an emergency caesarean [28]. As a result, it was found that external behavior of the team with professional appearance, an emphatic anesthetists and clear announcements reduce threat of the situation to the mother. It is conceivable that training of a procedure will liberate cognitive recourses of the team, to improve these factors. Experiences from resuscitation training—a situation with similar pressure of performance—show clearly, that team performance can be improved by team training [26]. One thesis could be that the quality of care for the awake women until anesthesia starts reduces the threat and the occurrence of posttraumatic stress disorder. We recommend a further development of hypotheses for relevant outcome parameters for the situation of emergency caesarean (e.g. posttraumatic stress disorder).

The reduction of reported possible adverse events could also become an outcome parameter for training. However, since reports are voluntary and anonymous, not every event automatically triggers a report.

There is an existing variety of different training concepts in the area of delivery rooms. However, the designation of the term interprofessional and multiprofessional remains unclear. It can stand for several professions and departments of one hospital are involved [29] or for example a complete obstetric collaborative network is trained [30]. Therefore, when assessing and comparing studies, the collective which has been trained and in which way must be taken into account. In Germany, currently there is no obligation for practical training in interprofessional delivery room teams. Nevertheless, there are various local projects, that differ in composition of training group, duration and goal setting. Skill training of procedures with actors [12], team training of physicians and midwifes using low fidelity simulation [13] and combinations of knowledge gain, practical skill- and non-technical skill training using high fidelity simulation over 2 days can be attended [11].

## Limitations

The sole number of trained employees in a real-life emergency situation cannot be used to prove the translation of training objectives. If, for example, the team leader of a real emergency cesarean is the only untrained person, this has a tremendous impact on extinction and confusion in task management. This study has the limitation of having a monocentric design. Changes e.g. in the safety culture of individual departments, structures of identifying patients at risk and personnel turnover could have an influence on the investigated topic.

## Conclusion

Training evaluation and transfer of knowledge provide a possibility to evaluate training up to level three of Kirkpatrick. Negative items for transfer should be considered more intensively in multidisciplinary teams. The presented training course was perceived as useful by the professionals and attitudes toward training were positive; and the content was positively reinforced in practice 1 year after training. To justify the training with outcome data of newborn, large case numbers are necessary, and a multicenter study design is recommended.

## Appendix 1

**Table:** Shares used from the questionnaires Training Evaluation Inventory [18] and Transfer Climate [19]

	German-speaking items	English items
Skala 1	Angenommener Nutzen des Training	Perceived usefulness
1	Ich finde das Simulationstraining nützlich für meinen Beruf	I find the simulation training useful for my job
2	Zeit in dieses Simulationstraining zu investieren war sinnvoll	Investing time in this simulation training was useful
3	Ich kann die Inhalte des Simulationstrainings in meinem Beruf anwenden	I can apply the content of this simulation training in my job
4	Ich ziehe persönlichen Nutzen aus diesem Simulationstraining	I derive personal use from this simulation training
Skala 2	Einstellung zum Training	Attitude towards training
1	Ich werde das Gelernte im beruflichen Alltag anwenden	I will apply what I learned to my day-to-day work
2	Ich finde es gut, dass die angesprochenen Themen vermittelt, bzw. besprochen wurden	I like that the themes were imparted and/or discussed
3	Ich würde das Simulationstraining meinen Kollegen empfehlen	I would recommend this simulation training to my colleagues
Skala 3	Soziale Wahrnehmung	Social Cues
1	Meine Kolleg*innen können mir zeigen, wie ich die Inhalte des Trainings in der Arbeit anwenden kann	My colleagues help me applying the concepts learned in the training at work
2	Mein Vorgesetzten geben mir die Möglichkeit, die Inhalte des Trainings in der täglichen Arbeit anzuwenden	Supervisors give me the chance to try out the training in the job immediately
Skala 4	Arbeitsrealität	Task Cues
1	In der Arbeit habe die Möglichkeiten mich so zu verhalten, wie ich es im Simulationszentrum gelernt habe	Jobs are designed so that I can do the job the way we were trained
2	Es sind ausreichen Ressourcen (Personen o.Equipment o. Zeit) vorhanden, um die Arbeit so zu erledigen, wie es in dem Simulationstraining vermittelt wurde	The equipment (staff, time, material) allows me to use the skills gained in training
Skala 5	Positive Verstärkung	Positive Reinforcement
1	Wenn ich die Inhalte des Simulationstrainings anwende, wird die Arbeit leichter	When I use the content of training, jobs are easier
2	Meine Vorgesetzten erkennen es positiv an, wenn ich die Inhalte der Trainings korrekt umsetze	Supervisors appreciate me when I do the job as taught in training
3	Meine Kollegen merken es positiv an, wenn ich meine Arbeit so ausführe, wie in dem Simulationstraining gelernt	Colleagues appreciate me when I do the Job as we were taught in training
Skala 6	Negative Verstärkung	Negative Reinforcement and Punishment
1	Kolleg*innen machen sich schon mal über die in dem Simulationstraining vermittelten Inhalten lustig	Colleagues ridicule the use of methods taught in training
2	Wenn ich die Inhalte des Simulationstrainings nicht anwende, werde ich ermahnt	When I fail to use the skills learned in training, I can expect to be reprimanded
3	Um meine Arbeit richtig auszuführen, muss ich mich genau an die Techniken und Verfahrensweisen halten, die ich in den Trainings erlernt habe	To do my job correct, I have to follow the techniques and procedures learned in training
Skala 7	Extinktion	Extinction
1	Für meine Vorgesetzten ist es eher nebensächlich, ob ich die Inhalte des Simulationstrainings anwende, solange die Arbeit erledigt wird	Supervisors do not care if I use the skills learned in training, as long as I get the job done
2	Ich habe wenige Gelegenheiten, die Inhalte der Simulationstrainings anzuwenden, so dass ich diese nicht vollständig verinnerlichen kann	***I ***have so little chance to use some of the skills learned since training, that I probably couldn´t perform them now
